# Expression of Connexins 37, 40 and 45, Pannexin 1 and Vimentin in Laryngeal Squamous Cell Carcinomas

**DOI:** 10.3390/genes14020446

**Published:** 2023-02-09

**Authors:** Ivan Mizdrak, Maja Mizdrak, Anita Racetin, Braco Bošković, Benjamin Benzon, Merica Glavina Durdov, Katarina Vukojević, Natalija Filipović

**Affiliations:** 1 Department of Otorhinolaryngology, Head and Neck Surgery, University Hospital of Split, Spinčićeva 1, 21000 Split, Croatia; 2 Department of Nephrology and Hemodialysis, University Hospital of Split, Šoltanska 1, 21000 Split, Croatia; 3 Department of Anatomy, Histology and Embryology, University of Split School of Medicine, Šoltanska 2, 21000 Split, Croatia; 4 Department of Pathology, Forensic Medicine and Cytology, University Hospital of Split, University of Split School of Medicine, Spinčićeva 1, 21000 Split, Croatia

**Keywords:** connexin 37, connexin 40, connexin 45, pannexin 1, vimentin, laryngeal squamous cell carcinoma, metastases, gap junction, hemichannel, immunofluorescence

## Abstract

Approximately 60% of patients with squamous cell carcinoma (LSCC) have regional occult metastatic disease/distant metastases at the time of diagnosis, putting them at higher risk for disease progression. Therefore, biomarkers are needed for early prognostic purpose. The aim of this study was to analyze the expression pattern of connexins (Cx) 37, 40 and 45, pannexin1 (Panx1) and vimentin in LSCC and correlate with tumor grade (G) and outcome. Methods: Thirty-four patients who underwent (hemi-)laryngectomy and regional lymphadenectomy due to LSCC from 2017 to 2018 in University Hospital Split, Croatia, were studied. Samples of tumor tissue and adjacent normal mucosa embedded in paraffin blocks were stained using the immunofluorescence method and were semi-quantitatively analyzed. Results: The expression of Cx37, Cx40, and Panx1 differed between cancer and adjacent normal mucosa and between histological grades, being the highest in well-differentiated (G1) cancer and low/absent in poorly differentiated (G3) cancer (all *p* < 0.05). The expression of vimentin was the highest in G3 cancer. Expression of Cx45 was generally weak/absent, with no significant difference between cancer and the controls or between grades. Lower Panx1 and higher vimentin expression were found to be prognostic factors for regional metastatic disease. Lower Cx37 and 40 expressions were present in patients with disease recurrence after the three-year follow-up period. Conclusion: Cx37 and Cx40, Panx1, and vimentin have the potential to be used as prognostic biomarkers for LSCC.

## 1. Introduction

Intercellular communication is a key factor in cellular function and tissue homeostasis and is controlled at intracellular, extracellular, and intercellular levels [1,2]. Intercellular communication is mainly mediated by gap junctions (GJs) at the cell membrane, formed by the interaction of two hemichannels (HCs) [3,4]. Each HC, also known as a connexon, is composed of six connexins (Cxs), which are defined as tetraspanning integral membrane proteins [5]. In humans, 21 connexin subtypes have been characterized, and while some of them are expressed in different tissue types, most Cxs exhibit a tissue-specific expression pattern [2,6]. They are named according to their molecular weight, from the smallest (Cx23) to the largest (Cx62) [7]. GJs enable the intercellular transport of ions and small and hydrophilic molecules with molecular weights less than 1–1.5 kilodaltons [2]. In addition, connexins provide a communication pathway between the intracellular and extracellular environments through the formation of hemichannels, which is important for autocrine/paracrine signal transduction [7]. They also have some channel-independent functions, including the activation of intracellular signaling pathways, adhesion between cells, and interaction with the cytoskeleton [7,8].

Following the malignant transformation of the cell, the dysregulation of Cxs may promote or suppress tumorigenesis and metastasis [9]. Regardless of the origin of the cancer, the expression of Cxs is often reduced [7]. According to the literature, members of the connexin protein family act as tumor suppressors, including Cx32 in various organs, Cx43 in breasts and lungs, and Cx26 in breasts [10,11]. The ability to act as a tumor suppressor can vary significantly depending on tissue type and cancer stage, as well as on the connexin isoform [10]. Some connexin isoforms may be pro-tumorigenic, promoting metastatic growth and treatment resistance in melanoma (Cx26) and brain tumors (Cx30 and Cx43) or causing an unfavorable prognostic effect in renal cancer patients (Cx37) [10,12]. The function of connexin inhibitory channels may overlap with that of pannexins [7]. Pannexins (Panx1, Panx2; Panx3) are a family of glycoproteins that form large pore channels at the cell membrane [13,14]. They are widely distributed in almost all cell types in physiological and pathological processes, including cancer [15,16,17]. Panx1 monomers oligomerize to form large hexameric plasma membrane channels that remain closed under physiological conditions, but open under pathological conditions [18]. Pannexins allow the passage of molecules up to 1 kDa in size that are important for paracrine and autocrine signaling, and also have intracellular functions, serving as calcium leak channels in the endoplasmic reticulum [13,15,19,20]. The Panx-1-dependent release of nucleotides contributes to their various functions, including cell clearance and inflammation, cancer progression, blood pressure regulation, metabolic defects, and neurological disorders [21]. Panx1 may be involved in the development of cancer in humans, although the exact pathophysiological mechanism is still unknown. In most cancer types, Panx1 expression negatively correlates with disease onset or progression, but some exceptions have been reported [9]. It is possible that Panx1 expression is necessary in early development, but needs to be downregulated in adulthood to avoid the negative effects on pathological conditions, including cancer [9].

Laryngeal squamous cell carcinoma (LSCC) is the most common head and neck tumor, with an overall prevalence of 1–2% out of all malignancies in humans [22]. Many patients have regional lymph nodes or distant metastases at the time of diagnosis, meaning that approximately 60% of patients are at an advanced stage (III or IV) [23]. Patients’ quality of life, based mainly on organ preservation, has improved in recent decades with multi-modal treatments, less invasive surgical techniques, and new oncologic therapies.

Despite this, LSCC remains one of the oncologic diseases for which the 5-year survival rate has not decreased significantly over the past 40 years (from 66% to 63%), although the overall incidence has decreased [22]. Possible explanations include local recurrence and the occurrence of distant metastases that do not respond to conventional treatments; the long-term benefit of an organ-preserving approach is also questionable [24]. Standardized diagnostic and clinical tools are unfortunately still unable to distinguish patients with hidden metastatic disease or those with a poor prognosis. These facts emphasize the need to search for new reliable prognostic biomarkers for this malignancy. The role of pannexins, Cx37, Cx40 and Cx45 in LSCC has not yet been explored. Therefore, the aim of this study was to analyze the expression of Cxs and Panx1 in cancer and adjacent normal mucosa and vimentin, an indirect marker of epithelial–mesenchymal transition in LSCC, and to correlate the results with pathological parameters, pathological stage and clinical outcome.

## 2. Materials and Methods

### 2.1. Subjects and Study Protocol

**Figure 1:**
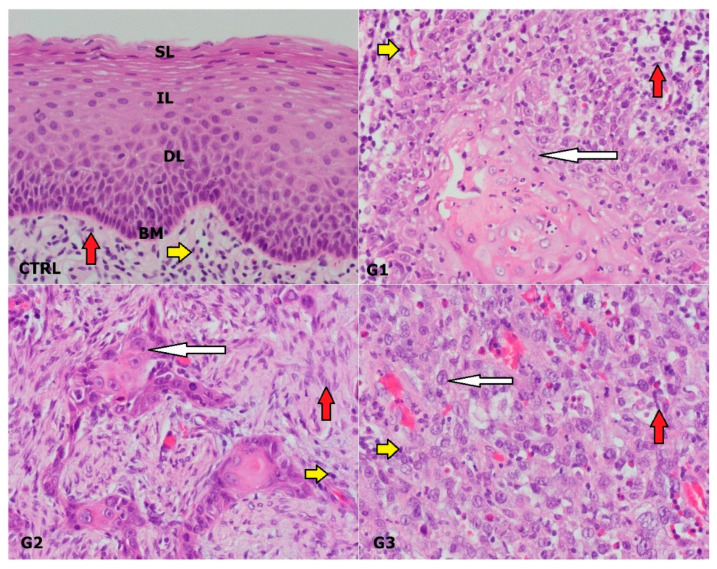
Immunohistochemical presentation of different Laryngeal squamous cell carcinoma (LSCC) grades and normal laryngeal mucosa; H & E, G1—grade 1, G2—grade 2, G3—grade 3; CTRL—control group; SL—superficial layer of normal laryngeal epithelium, IL—intermediate layer, DL—deep layer, BM—basal membrane, red arrow—fibroblast, white arrow—malignant cell; yellow arrow—lymphocyte. Scale bar = 20 µm, refers to all images (magnification 400×).

Thirty-two patients who underwent partial or total laryngectomy for LSCC at the Department of Otolaryngology and Head and Neck Surgery, University Hospital of Split, Split, Croatia, between 1 January 2017 and 31 December 2018, were included in this retrospective study. Patients who had undergone primary oncologic treatment and patients with distant metastases were excluded from the study. Paraffin blocks of laryngeal tumor tissue and adjacent normal mucosa were obtained from the Department of Pathology, Cytology and Forensic Medicine of the same institution, and clinical data were collected from the hospital records. For each patient, the following data of interest were recorded: age, sex, tumor location, type of surgery, tumor size, TNM staging, lymphovascular and perineural invasion and pathohisto-logical grade (Figure 1). Immunofluorescence staining was performed at the Department of Anatomy, Histology and Embryology, University of Split, School of Medicine. For every patient, both malignant and normal laryngeal tissues were discriminated by an experienced pathologist and simultaneous staining was performed. The expression of connexin 37, connexin 40, connexin 45, pannexin-1 and vimentin was analyzed in the tumor samples in comparison to the adjacent healthy tissue by two independent investigators.

### 2.2. Immunofluorescence

Five µm thin slides were cut from the paraffin blocks, deparaffinized in xylene, dehydrated through grades of alcohol, washed in distilled water, boiled for 20′ in sodium citrate buffer (pH = 7) at 95 °C in a water steamer, then gradually cooled to room temperature and rinsed with phosphate-buffered saline (PBS). Protein blocking buffer (ab64226, Abcam, Cambridge, UK) was then applied for 30 min to prevent non-specific staining. The primary antibodies (Table 1) were then applied and incubated overnight in a humidity chamber. The next day, after washing in PBS, the samples were rinsed with PBS, before incubation with the appropriate secondary antibodies (Table 1) for 60 min. After washing in PBS, DAPI (4′,6-diamidino-2-phenylindole) was applied for 2′ blue nuclear staining, and the slides were washed and coverslipped (Immu-Mount, Thermo Shandon, Pittsburgh, PA, USA). No staining was observed when the primary antibodies were omitted from the immunofluorescence protocol.

### 2.3. Quantification and Statistical Analysis

Microscopic analysis was performed with the Olympus IX51 inverted fluorescence microscope (Olympus, Tokyo, Japan) with a 40× objective and 10 non-overlapping fields, using CellA Imaging Software for Life Sciences Microscopy (Olympus Soft Imaging Solution GmbH, Münster, Germany). Photographs were processed using Image J software (National Institutes of Health, Bethesda, MD, USA). Semiquantitative analysis of the expression of connexins, pannexin-1, and vimentin in normal laryngeal and tumor tissues was performed. We used the immunoreactivity score (IRS) classification, multiplying the percentage of positive cells and the intensity of staining, as previously described by others [25]. The percentage of positive cells ranged from 0 to 4, where 0 = no positive cells, 1 ≤ 10% positive cells, 2 = 10–50% positive cells, 3 = 50–80% positive cells, 4 ≥ 80% positive cells and the intensity of staining scores ranged from 0 to 3, where 0 = no color reaction, 1 = mild reaction, 2 = moderate reaction and 3 = strong reaction. The calculated result was expressed by 0–12 points, i.e., 0–1 = negative (IRS 0 = negative), 2–3 = mild (IRS 1 = positive, weak expression), 4–8 = moderate (IRS 2 = positive, moderate expression); 9–12 = strongly positive (IRS 3 = positive, strong expression).

Statistical analysis was performed using the National Council for the Social Studies (NCSS) 2022 software (Kaysville, Utah, USA). Statistical significance was set at *p* < 0.05, and all confidence intervals (CI) were at the 95% level. Spearman’s correlation coefficient (Rho) was used to calculate the degree of association. Analysis of the statistical significance of the differences in several numeric variables was performed with the Kruskal–Wallis test and analysis of the differences between the two groups was performed with the Mann–Whitney and Dunn tests. The Chi2 test was used for the analysis of the categorical variables. A logistic regression model was performed to determine the independent prognostic factors. Receiver-operating characteristic curves (ROC) were constructed to calculate the cut-off values that were significant for both recurrence and overall survival.

## 3. Results

### 3.1. Clinical and Pathological Characteristics of the Study Sample

Among the 32 patients with LSCC, 29 (91%) males and 3 females (9%) with a mean age of 68 (min–max: 43–84) years. Patients were divided into three groups according to the degree of tumor differentiation (G1, G2; G3). There were 10 (31%), 12 (38%), and 10 (31%) patients in each group, respectively. The clinical and pathohistological characteristics are summarized in Table 2.

Patients with different tumor grades did not differ by age, sex, or tumor volume (all *p* > 0.05). Lymphovascular invasion was more common in poorly differentiated LSCC patients (G3) (*p* = 0.049).

### 3.2. Expression of Cx37

**Figure 2:**
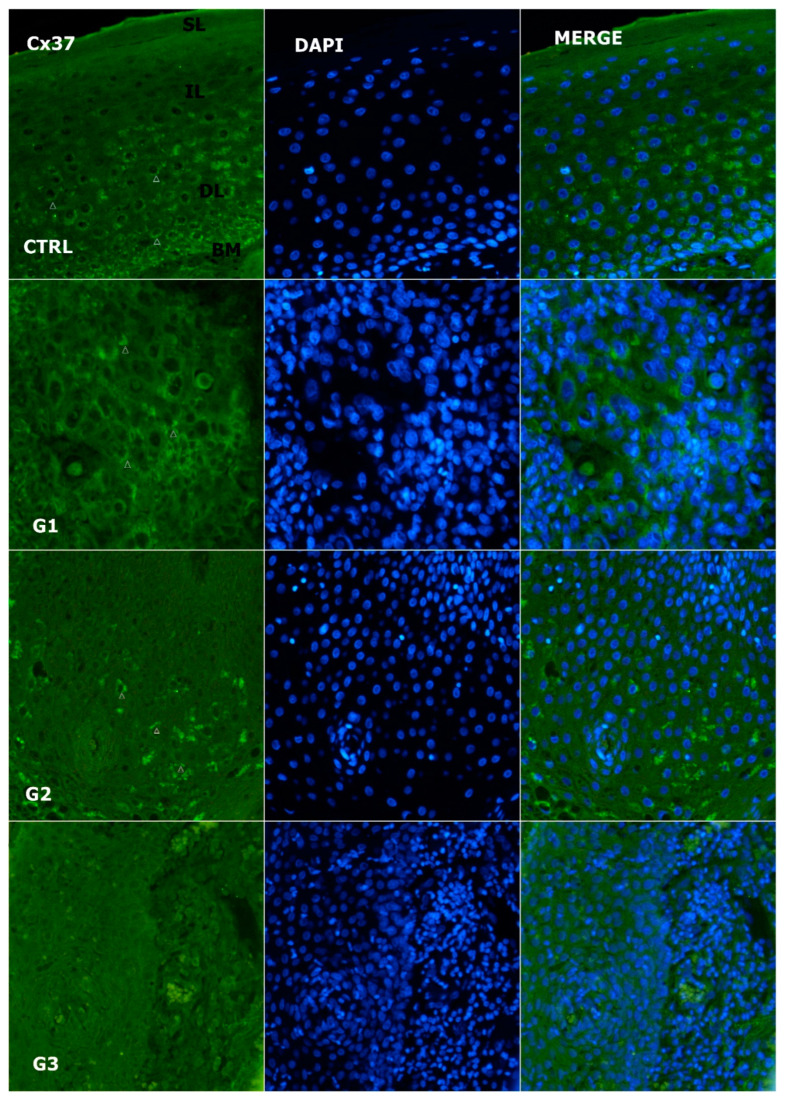
Expression pattern of connexion 37 (Cx37; arrows) in normal laryngeal mucosa and different grades of LSCC (green—Cx37, blue—DAPI, G1—grade 1, G2—grade 2, G3—grade 3, CTRL—control group, SL—superficial layer of normal laryngeal epithelium, IL—intermediate layer, DL—deep layer; BM—basal membrane). Scale bar = 20 µm, refers to all images (magnification 400×).

**Figure 3:**
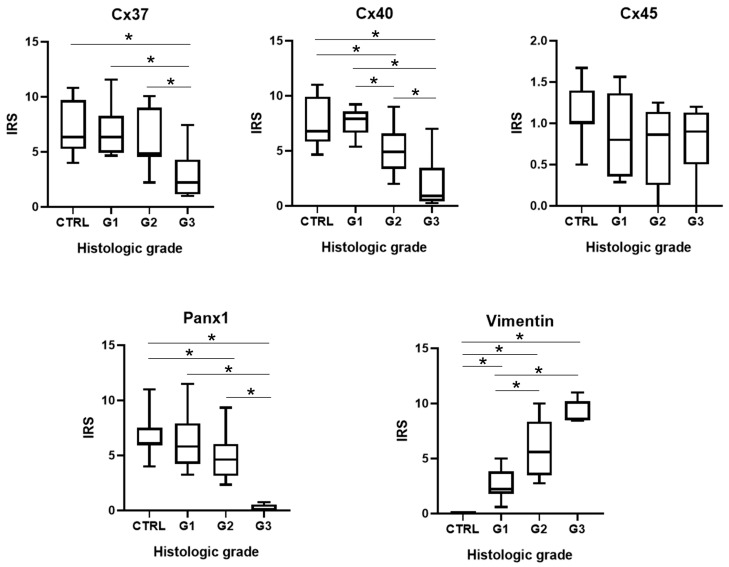
Comparison of medians (IQR) of immunoreactivity staining score (IRS) according to different pathohistological grades (G) and the control group (CTRL) for connexins 37, 40 and 45 (Cx37, Cx40, Cx45), pannexin 1 (Panx1) and vimentin. Asterisk—significant differences between groups (*p* < 0.05).

Immunohistochemically, Cx37 was granularly stained on the plasma membrane of epithelial cells. Its expression was different in the tumors and adjacent normal laryngeal mucosa (Figure 2 and Figure 3) (*p* = 0.016, χ^2^ = 8.294). The median IRS in the different histological grades was 5 (IQR: 4.14–7.69), and 6.34 (IQR: 5.27–9.71) in the control. In the mucosa of the larynx, strong Cx37 expression was found mainly in the deeper layer of the epithelium. Cx37 expression was highest in cancer stage 1 patients and lowest in cancer stage 3 patients. There was no difference in median IRS between stages 1 and 2, with values of 6.36 (IQR: 4.93–8.27) and 4.94 (IQR: 4.59–8.96), respectively (*p* = 0.308). Cx37 expression was significantly different between grade 1 and 2 compared with grade 3, with values of 2.21 (IQR: 1.15–4.29), *p* = 0.015, Z = 2.440, and *p* = 0.017, Z = 2.388, respectively. Cx37 expression was significantly different between G3 and the control (*p* = 0.005; Z = 2.838). Tumor Cx37 expression did not correlate with vimentin expression (*p* = 0.403) or Cx45 expression (*p* = 0.391). However, there was a positive correlation between Cx37 and Panx1 expression (*p* = 0.037; Rho = 0.411) and Cx37 and Cx40 expression (*p* < 0.001; Rho = 0.619). 

### 3.3. Alteration of Expression of Cx40

**Figure 4:**
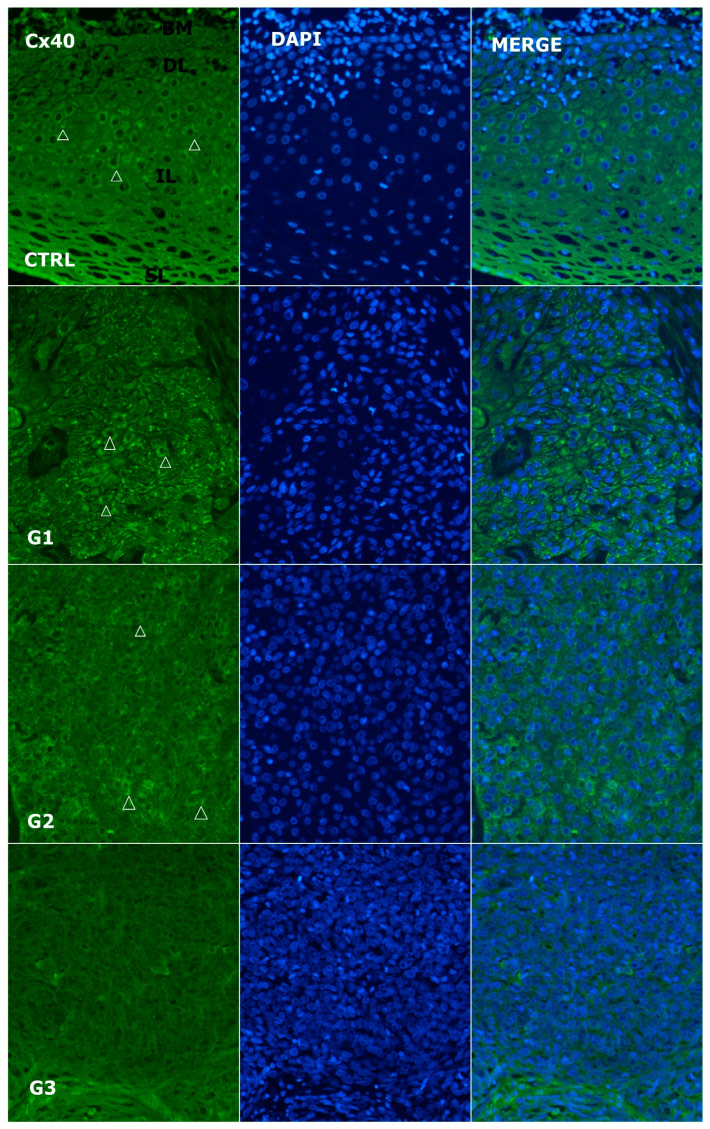
Expression pattern of connexin 40 (Cx40; arrows) in normal laryngeal mucosa and different grades of LSCC (green—Cx40, blue—DAPI, G1—grade 1, G2—grade 2, G3—grade 3, CTRL—control group, SL—superficial layer of normal laryngeal epithelium, IL—intermediate layer, DL—deep layer; BM—basal membrane). Scale bar = 20 µm, refers to all images (magnification 400×).

Immunohistochemically, Cx40 expression differed in the tumors and adjacent normal laryngeal mucosa (Figure 3 and Figure 4) (*p* < 0.001, χ^2^ = 15.155). The median IRS was 5.44 (IQR: 2.13–7.47) and in the control class, it was 6.79 (IQR: 5.83–9.93). In normal laryngeal mucosa, Cx40 was strongly expressed mainly in the plasma membrane of the middle/deep epithelial layer. Cx40 expression was reduced in less differentiated tumors. There was a difference in the median IRS between G1 and G2 (*p* = 0.009, Z = −2.629), with values of 7.89 (IQR: 6.65–8.59) and 4.91 (IQR: 3.34–6.56), respectively. Cx40 expression in G3 was weak, with a value of 0.57 (IQR: 0.43–2.25), and significantly lower than in G1 and G2, where *p* = 0.002, Z = 03.069 and *p* = 0.009, Z = 2.578, respectively. Comparison of Cx40 between the tumor and control grades showed significant differences between G2 and the control (*p* = 0.013; Z = 2.495) and G3 and the control (*p* = 0.002; Z = 3.059). However, there was no difference in Cx40 expression between G1 and the control (*p* = 0.659). There was a strong negative correlation between Cx40 and vimentin expression (*p* = 0.002; Rho = −0.708).

### 3.4. Alteration of Expression of Cx45

**Figure 5:**
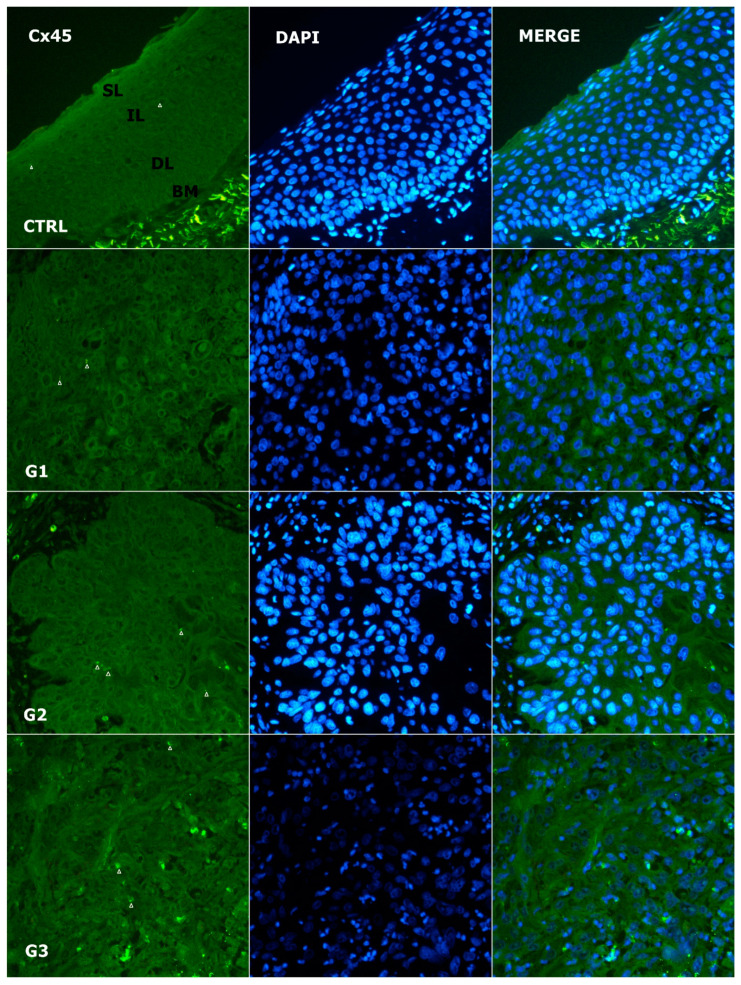
Expression pattern of Cx45 (arrows) in normal laryngeal mucosa and different grades of LSCC (green—Cx45, blue—DAPI, G1—grade 1, G2—grade 2, G3—grade 3, CTRL—control group, SL—superficial layer of normal laryngeal epithelium, IL—intermediate layer, DL—deep layer; BM—basal membrane). Scale bar = 20 µm, refers to all images (magnification 400×).

As shown in Figure 3 and Figure 5, the expression of Cx45 was also membranous, but absent/weak in the tumor and control, with values of 0.83 (IQR: 0.37–1.16) and 1 (IQR: 1–1.14), respectively. No significant difference was found between the tumors and controls or between tumor grades (*p* = 0.373 and *p* = 0.670, respectively). There was no correlation with the expression of vimentin (*p* = 0.342), Cx40 (*p* = 0.881), or Panx1 (*p* = 0.708).

### 3.5. Alteration of Expression of Panx1

**Figure 6:**
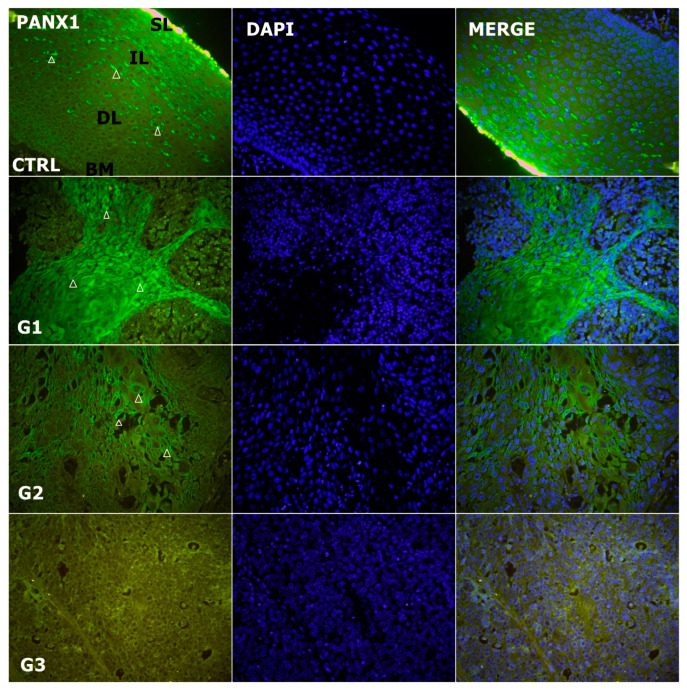
Expression pattern of pannexin 1 (Panx1; arrows) in normal laryngeal mucosa and different grades of LSCC (green—Panx1, blue—DAPI, G1—grade 1, G2—grade 2, G3—grade 3, CTRL—control group, SL—superficial layer of normal laryngeal epithelium, IL—intermediate layer, DL—deep layer; BM—basal membrane). Scale bar = 20 µm, refers to all images (magnification 400×).

Immunohistochemically, Panx-1 was granularly stained in the cell membrane. Panx-1 expression differed in the tumors and adjacent normal laryngeal mucosa (Figure 3 and Figure 6) (*p* < 0.001, χ^2^ = 19.672). The median IRS was 4.27 (IQR: 2.71–6.33) and 6 (IQR: 6–7.5) in the controls. In normal laryngeal tissue, Panx-1 was highly expressed and was predominantly located in the superficial/middle layer of the epithelium. In tumor cells, Panx-1 was the highest in G1 and lowest in G3. There was no difference in the median IRS between G1 and G2 (*p* = 0.386), which was 5.81 (IQR: 4.25–7.92) and 4.62 (IQR: 3.15–6.05), respectively. The expression of Panx-1 in G3 was almost completely absent, with a median value of 0 (IQR: 0–0.56), which was significantly lower than in G1 and G2, where *p* < 0.001, Z = −3.784 and *p* < 0.001, Z = −3.919, respectively. When Panx-1 was compared between the tumor and control groups, differences were observed between G2 and the control (*p* = 0.026; Z = −2.221) and G3 and the control (*p* < 0.001; Z = −3.997). There was no difference between G1 and the control (*p* = 0.344). Expression of Panx-1 correlated positively with the expression of Cx40 (*p* = 0.037; Rho = 0.411) and negatively with the expression of vimentin (*p* = 0.045; Rho = −0.492).

### 3.6. Expression Pattern of Vimentin

**Figure 7:**
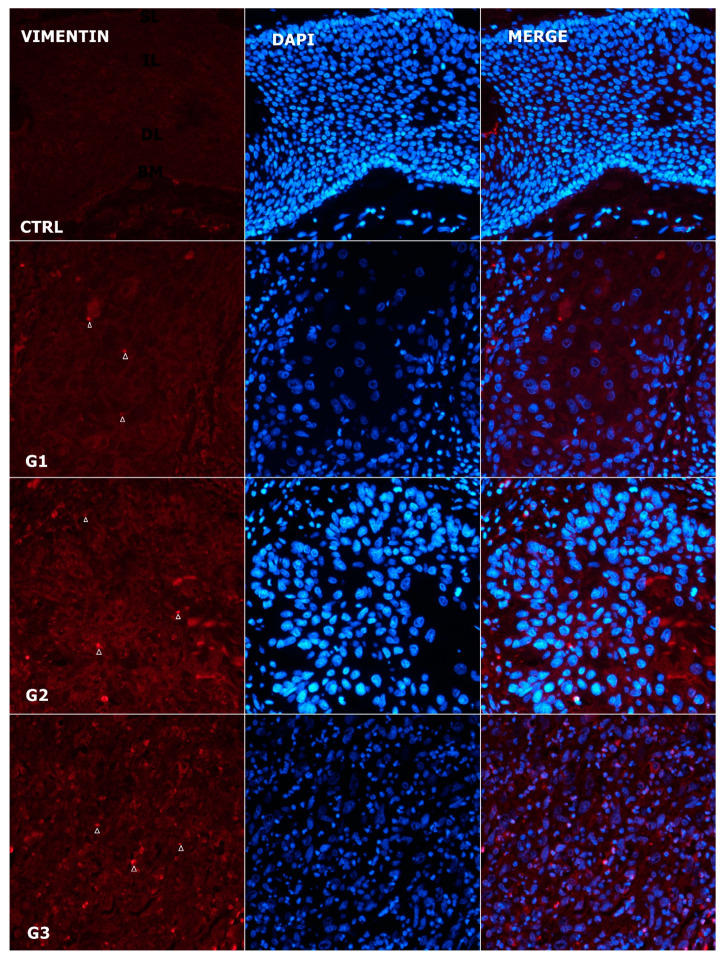
Expression pattern of vimentin (arrows) in normal laryngeal mucosa and different grades of LSCC (red—vimentin, blue—DAPI, G1—grade 1, G2—grade 2, G3—grade 3, CTRL—control group, SL—superficial layer of normal laryngeal epithelium, IL—intermediate layer, DL—deep layer; BM—basal membrane). Scale bar = 20 µm, refers to all images (magnification 400×).

The expression of vimentin in normal laryngeal mucosa was negative (Figure 3 and Figure 7). Homogeneous cytoplasmic expression was found in poorly differentiated laryngeal cells. It was the lowest in G1, with a median value of 2.2 (IQR: 1.8–3.83), and the highest in G3, with a median value of 8.6 (IQR: 8.515–10.2). A statistically significant difference was observed between vimentin expression when comparing G1 vs G2 (median 5.6; IQR: 3.48–8.36), where *p* = 0.035, Z = −2.111, and when comparing G1 vs G3, when *p* = 0.006, Z = −2.761, respectively. There was no difference in vimentin expression between G2 and G3, where *p* = 0.094. The analysis of vimentin expression in the control and tumor tissues showed a statistically significant difference for all tumor grades (all *p* < 0.05). In contrast to the expression of hemichannel proteins, which showed a wreath-shaped pattern, vimentin-positive tumor cells exhibited an intracellular distribution.

### 3.7. Alteration in Expression Pattern of Cx37, Cx40, C45, Panx1 and Vimentin in Comparison to Pathological Characteristics

The analyzed markers are related to pathological parameters. Tumor size (median volume: 7.81 (min–max: 0.25–48 cm^3^)) did not correlate with Cx37 (*p* = 0.326), Cx40 (*p* = 0.893), Cx45 (*p* = 0.652), or Panx1 (*p* = 0.579). However, there was a positive correlation between tumor size and vimentin expression (*p* = 0.029; Rho = 0.563). Moreover, the median level of Panx1 IRS was 13-fold higher in tumors without lymphatic invasion (LVI) (*p* = 0.007). In contrast, vimentin expression was 4-fold higher in tumors with LVI (*p* = 0.018). There was no correlation between LVI and the expression of Cx37, Cx40, and Cx45 (all *p* > 0.05). Perineural invasion (PNI) was absent in 94% of cases and correlated with the expression of Cx40, Cx45, Panx1, and vimentin. Cx40 expression was higher in cases with PNI (*p* < 0.001). Conversely, the expression of Cx45, Panx1, and vimentin was higher in cases without PNI (all *p* < 0.001). Tumor location was not associated with Cxs or Panx-1 (all *p* > 0.05). Vimentin expression was the highest in transglottic tumors (*p* = 0.029) and higher in supraglottic compared with glottic tumors.

### 3.8. Diagnostic and Prognostic Role of Cx37, Cx40, Cx45, Panx1 and Vimentin

All patients were without distant metastases at the time of diagnosis. N status was analyzed according to the presence (N+ including N1–N3) or absence (N0) of regional lymph node metastases. There was no difference in connexin expression according to N status (all *p* > 0.05). The Panx-1 IRS median was significantly higher in patients with a negative neck status compared to metastatic neck disease, with values of 4.5 and 2 (*p* = 0.045). Vimentin expression was higher in patients with a positive N status (median 7.65 versus 3.83), where *p* = 0.048. According to the logistic regression model, Panx-1 was an independent prognostic factor for regional metastatic disease in LSCC (*p* = 0.049, 95% CI: 0.563–0.980, OR: 0.76; regression coefficient −0.271). A higher histological grade, higher T and positive lymphovascular invasion were also proven to be risk factors for positive neck disease (all *p* < 0.05). In addition, positive neck disease was an independent risk factor for disease recurrence and mortality (*p* = 0.021 and *p* < 0.001, respectively). The expression of Cx37, as well as Cx40, was stronger in patients who had recurrent disease in a follow-up period of 3 years (*p* = 0.044 and *p* = 0.024, respectively). The median IRS values of Cx37 and Cx40 were 3.25 (IQR: 2.06–4.53) and 0.57 (IRS: 0.44–4.78). In patients without a recurrence medians of the Cx37 and Cx40 expression were 5.5 (IRS: 4.67–8.83) and 6 (IRS: 3.23–7.89), respectively. 

## 4. Discussion

In our study, we investigated the expression of various connexins and Panx-1 as possible prognostic markers in LSCC. The expression of Cx37, Cx40, Panx-1, and vimentin differed significantly between the epithelium of normal laryngeal mucosa and the atypical epithelial cells of LSCC and was related to the histological grade and biological behavior of the cancer. The main function of connexins and Panx1 is to exchange information between cells [15]. Cross-talk between tumor cells and their environment enables cancer cells to reprogram the tumor microenvironment and migrate to distant sites, which promotes angiogenesis, immune surveillance, EMT, invasion, spread, and resistance to therapy [1]. To our knowledge, this is the first study to investigate the immunohistochemical expression of pannexin-1, connexin 37, 40, and 45 in human LSCC and laryngeal tissue, compared with vimentin and pathological parameters and outcomes. The highest staining intensity of hemichannel proteins was observed in well-differentiated tumors and gradually decreased, until they completely disappeared in poorly differentiated/undifferentiated LSCC. Vimentin is an intermediate type III filament, a structural protein of the cytoskeleton and viscoelastic scaffold [26]. Under pathological conditions, vimentin modulates genes for EMT inducers, such as Snail, Slug, Twist, and ZEB1/2, as well as epigenetic factors [26]. Vimentin suppresses cell differentiation, regulates pluripotent potential, and increases the stemness of cancer cells, promoting tumor invasiveness and causing resistance to therapy [26,27]. According to Usman et al., vimentin is the “heart” of EMT-mediated metastasis [26]. Recent evidence on the role of vimentin in LSCC has shown that vimentin plays an important negative prognostic role in LSCC, regardless of the TNM stage and histological grade of the tumor [28].

In contrast to vimentin, the possible involvement of Cxs and Panxs in LSCC progression remains unexplored, and the elucidation of their control mechanisms offers an opportunity to target cancer behavior [7]. To date, connexins 26, 30, 31.1, 32, and 43 have been studied in LSCC [29]. Immunohistochemical results have shown no change in the expression of Cx26, 30, 32, and 43 during laryngeal epithelial carcinogenesis [30], but Cx26 and Cx40 were found to play a role in tumor suppression [29,31]. Many studies have reported a correlation between a decrease in connexin expression and an increase in histological grade or dislocation of connexin within the cell [32]. The most important question remains whether connexins have tumor-suppressive or tumor-promoting effects [9]. Experts in this field suggest that altered connexin activity may be a unique feature of cancer [32]. The evaluation of connexins in cancer involves the study of GJ-related, hemichannel, and non-channel functions in the context of cell progression and checkpoint control, cancer cell proliferation and differentiation, metabolic reprogramming, angiogenesis, cancer microenvironments, tissue invasion and metastasis, and various cancer pathologies and therapeutic potential [9]. In a review paper, Jiang summarized the current knowledge on the tumor suppressive role of connexins in the early stages of cancer progression or in primary tumors, while noting how they play an opposite role in late or advanced cancers and metastases [9]. Moreover, Mulkearns-Hubert in his review article highlights the tumor suppressive or pro-tumorigenic effect on various human malignancies [32].

According to our results, the expression of Cx37, Cx40, and Panx-1 might be abolished in less differentiated cancer. We hypothesize that the lower expression of Cx37, Cx40, and Panx-1 and the higher expression of vimentin may be associated with a more aggressive tumor phenotype, possibly by regulating growth and tumorigenic properties. Moreover, this may confirm the hypothesis of the tumor-suppressive activity of Panx-1 reported in the C6 glioma cell line, adenocarcinoma of gallbladder and basal and squamous cell carcinoma of skin [9]. The proposed antitumorigenic mechanisms of Panx1 reduce cell proliferation, cell motility, anchorage-independent growth, and tumor growth in experimental animals [33]. The immunohistochemical study of Panx-1 with Ki-67 in human gallbladder and gallbladder adenocarcinomas showed that Panx1 expression negatively correlated with proliferation in gallbladder carcinomas [34]. In non-melanoma human skin cancers, the downregulation of Panx1 may indicate its protective function against keratinocyte transformation [35]. In contrast, most of the published work suggests that the amplification or upregulation of Panx1 plays a pro-tumorigenic role [23]. Panx1 expression positively correlates with the occurrence or progression of melanoma, hepatocellular carcinoma, breast cancer, colorectal cancer, and hematologic malignancies [9]. Pannexins play distinct roles in different subtypes of cancer cells. Cells that express mutant Panx1 have a survival advantage in metastasis because ATP released through the pannexin channel acts on purinergic receptors to suppress apoptosis and reduce cell death, suggesting the alteration of tumor cell proliferation and disease progression through transmembrane signaling of solutes, such as nucleotides and Ca^2+^ [35].

Risk factors associated with an unfavorable outcome in LSCC include male gender, malnutrition, fitness status, alcohol and tobacco abuse, supraglottic location, perineural and lymphatic invasion, and a higher pathological stage and histological grade [36]. Thus, patients with supraglottic SCC have the highest frequency of regional metastases, both clinically visible and occult, due to its rich network of lymphatic vessels. Despite the availability of higher resolution laryngoscopes and a greater number of radiological images, clinical staging is insufficient, as 7–37% of patients with a clinical N0 stage still have occult metastatic lymph nodes [29]. Therefore, the identification of new biomarkers as disease predictors is needed [29]. Despite the promising results of several previously used biomarkers, such as E-cadherin, Ki-67, and Bcl-2, prospective studies are needed in systematic reviews to confirm the results in large numbers and laryngeal subsites [37,38,39]. Neither clinical, radiological nor histological evaluation have been included in the standard guidelines for the diagnosis and prognosis of LSCC [11,40].

In our study, the change in the expression pattern of Cx37, Cx40, pannexin-1, and vimentin was not only associated with different pathological stages (suggesting their possible role in tumor suppression), but also had a significant diagnostic/prognostic role. Indeed, lower Panx-1 expression was an independent prognostic factor for regional metastatic disease at diagnosis. Neck nodal status is a modifiable risk factor, which means that depending on a patient’s Panx-1 status, elective neck dissection may lead to a better prognosis. In addition, low or absent Cx37 and Cx40 expression in a 3-year follow-up period proved to be a marker for disease recurrence. This may be particularly important in the modern oncological era with more organ-preserving approaches in the removal of all tumors, including LSCC, with the main goal of maintaining/improving patients’ quality of life. This approach contributes to the possibility of overlooking occult metastatic disease, and thus an unfavorable outcome.

The beneficial or detrimental role of connexin and pannexin-1 in different cancer types remains controversial. Further research is needed to confirm whether the low expression or absence of connexin and pannexin-1 in undifferentiated LSCC, which have the worst prognosis, is a consequence or cause of the pathophysiological cascade, and why they exhibit opposite biological behavior in different tumors or even in different cell lines of the same tumor type, as reported in gliomas [9].

The main drawback of our study is the relatively small number of sample cohorts. Future studies, using other protein indices associated with the occurrence of squamous cell carcinoma, larger cohorts and in vitro approaches are needed in order to determine the exact mechanism of action between different grades of LSCC.

## 5. Conclusions

The recently discovered Pannexin-1 and the family of connexins have different roles in physiological and pathological conditions that have not yet been fully elucidated. According to studies, connexins and Panx1 are involved in the development of many human cancers. In our study, the expression of Cx37, Cx40, and Panx1 was investigated for the first time in surgically removed squamous cell carcinoma of the larynx by immunofluorescence method. We found a difference in the expression of Cx37 and Panx1 in cancer and adjacent normal laryngeal mucosa and between different histological grades of cancer. The intensity of staining was lowest in poorly differentiated LSCC (G3) and correlated with cancer recurrence after 3 years. These results support the idea that loss of cellular communication may be involved in the dedifferentiation of LSCC and suggest their potential utility as diagnostic and prognostic markers; further research is needed.

## Figures and Tables

**Table 1:** Primary and secondary antibodies used.

Antibodies		Host	Dilution	Source
Primary	Anti-Cx 37/GJA4 ab 181701	Rabbit	1:100	Abcam (Cambridge, UK)
	Anti-Cx40/ GJA5 ab213688	Rabbit	1:100	Abcam (Cambridge, UK)
	Anti-Cx 45/GJA7 ab135474	Rabbit	1:100	Abcam (Cambridge, UK)
	Anti-pannexin 1/PANX1	Rabbit	1:300	Temecula California 92590 ABN 242
	Anti-vimentin ab11256	Goat	1:300	R & D Systems, Minneapolis, MN, USA
Secondary	Anti-Rabbit IgG Alexa Fluor 488 711-545-152	Donkey	1:400	Molecular Probes Life Technologies, Eugene, OR, USA
	Anti-Sheep IgG Rhodamine Red 713-295-003	Donkey	1:400	Jackson Immuno Research Laboratories, Inc. (Baltimore, PA, USA)

**Table 2:** Clinical and pathohistological data of 32 patients with laryngeal squamous cell carcinoma according to histological grade.

Parameter		Grade 1 N = 10	Grade 2 N = 12	Grade 3 N = 10	*p*
Age		67.5 (51–84)	67 (47–82)	68 (43–80)	0.996 *
Sex	Male	9	11	9	0.177 **
	Female	1	1	1	
Localization of cancer	Glottic	8	3	1	0.012 **
	Supraglottic	1	2	2	
	Transglottic	1	7	7	
Tumor volume Median (min–max)	cm^3^	3.455 (0.280–36)	6 (0.245–10.500)	5 (2–48)	0.540 *
Lymphovascular invasion	yes	0	4	8	0.049 **
	no	10	8	2	
TNM	T1	4	1	0	0.003 **
	T2	3	2	0	
	T3	2	9	5	
	T4	1	0	5	
	N pos.	9	8	5	0.047 **
	N neg.	1	4	5	
	M neg.	10	12	10	0.031 **
	M pos.	0	0	0	
Stage	Early (I + II)	7	3	0	0.001 **
	Advanced (III + IV)	3	9	10	

* Kruskal–Wallis test; ** χ^2^ test.

## Data Availability

Data are available from the corresponding author upon reasonable request.
